# Race and Ethnicity and Early Do Not Attempt Resuscitation Orders After In-Hospital Cardiac Arrest

**DOI:** 10.1001/jamanetworkopen.2025.53504

**Published:** 2026-01-13

**Authors:** Caroline Raymond-King, Xunyun Wan, Ryan Cook, Gail D’Onofrio, Lauren Raymond-King, Paul Chan, Sarah M. Perman

**Affiliations:** 1 Department of Emergency Medicine, Yale University, New Haven, Connecticut; 2 Department of Biostatistics, Yale University, New Haven, Connecticut; 3 Department of Medicine, Section of Addiction Medicine, Oregon Health & Science University, Portland; 4 Department of Surgery, Yale University, New Haven, Connecticut; 5 Division of Cardiology, Department of Internal Medicine, St Lukes Health System & Mid-America Heart Institute, Kansas City, Missouri

## Abstract

**Importance:**

Black and Hispanic patients have lower survival rates for in-hospital cardiac arrest (IHCA) than White patients. Whether this is because do not attempt resuscitation (DNAR) orders for successfully resuscitated patients with IHCA are variable among different races and ethnicities remains unknown.

**Objective:**

To understand whether American Indian or Alaskan Native, Black, or Hispanic patients have different rates of early DNAR orders compared with White patients, and to examine whether survival differences by race and ethnicity persist among patients with early entry of DNAR orders.

**Design, Setting, and Participants:**

This prospective cohort study used data from the American Heart Association’s Get With the Guidelines – Resuscitation database, which includes IHCA data from more than 350 hospitals in the US from 2018 to 2013. Patients were aged at least 18 years, experienced an index IHCA, and were successfully resuscitated while on an admitted unit. Data were analyzed from September 26, 2024, through February 8, 2025.

**Exposure:**

IHCA.

**Main Outcomes and Measures:**

Main outcomes were associations of race and ethnicity with entry of early DNAR orders and, among patients early DNAR orders, the associations of race with survival to hospital discharge.

**Results:**

From 2018 to 2023, 93 843 patients (25 386 patients [27.1%] aged 60-69 years; 56 533 [60.2%] male) achieved ROSC after IHCA, including 2380 American Indian or Alaska Native patients (2.5%), 764 Asian patients (0.8%), 21 261 Black patients (22.7%), 6998 Hispanic patients (7.5%), and 56 989 White patients (60.7%). Overall, 25.3% and 37.4% of White patients had DNAR orders at 12 hours and 72 hours, respectively, compared with 21.3% and 33.4% of American Indian or Alaska Native patients, 21.4% and 32.7% of Black patients, and 22.2% and 33.2% of Hispanic patients. Compared with White patients, American Indian or Alaska Native, Black, and Hispanic patients were less likely to have DNAR orders entered within 12 hours (American Indian or Alaska Native: odds ratio [OR], 0.78 [95% CI, 0.67-0.91]; Black: OR, 0.74 [95% CI, 0.69-0.79]; Hispanic: OR, 0.90 [95% CI, 0.82-0.99]) or within 72 hours (American Indian or Alaska Native: OR. 0.86 [95% CI, 0.76, 0.98]; Black: OR, 0.73, [95% CI, 0.69-0.77]; Hispanic: OR, 0.89 [95% CI, 0.83, 0.97]). A total of 813 American Indian or Alaska Native patients (34.2%), 7168 Black patients (33.7%), and 2417 Hispanic patients (34.5%) with return of spontaneous circulation survived to discharge, compared with 22 226 White patients (39.0%). In adjusted analyses, among patients with an early DNAR order entered before 72 hours, there was no significant difference in survival to hospital discharge compared with White patients.

**Conclusions and Relevance:**

In this cohort study of patients successfully resuscitated from IHCA, American Indian or Alaska Native, Black, and Hispanic patients were less likely to have early DNAR orders than White patients. There were no differences in survival among patients with early DNAR orders placed.

## Key Points

**Question:**

Do American Indian or Alaskan Native, Black, or Hispanic patients have different rates of early do not attempt resuscitation (DNAR) orders compared with White patients after in-hospital cardiac arrest?

**Findings:**

In this cohort study including 93 843 patients from more than 350 hospitals, American Indian or Alaska Native, Black, and Hispanic patients were less likely to have early DNAR orders entered, compared with White patients; those with early orders had no difference in survival to discharge compared with White patients.

**Meaning:**

This cohort study found that after in-hospital cardiac arrest, American Indian or Alaska Native, Black, and Hispanic patients were less likely to have early DNAR orders than White patients, and there were no differences in survival among patients with early DNAR orders placed.

## Teaser

This cohort study examines whether American Indian or Alaskan Native, Black, or Hispanic patients had different rates of early do not attempt resuscitation (DNAR) orders compared with White patients and whether survival differences by race and ethnicity persisted among patients with early entry of DNAR orders.

## Introduction

Nearly 300 000 people experience in-hospital cardiac arrest (IHCA) annually in the US.^1^ Black patients who experience IHCA are less likely to survive than White patients.^2^ Differences by race persist even after initial resuscitation; Black patients who achieve return of spontaneous circulation (ROSC) after IHCA are still less likely to survive to hospital discharge than White patients.^2^

One modifiable, yet underexplored, determinant of survival after IHCA is early do not attempt resuscitation (DNAR) order placement; early order placement may lead to limitations on postarrest critical care in patients with survivable illness.^3^ Previous research has found that 20% of patients who survive IHCA have decisions for DNAR status made early, or within 72 hours of ROSC.^3,4^ Researchers have shown that DNAR orders often do not correlate with objective scores of survival with good neurologic outcome, and even patients with the best prognosis still have DNAR orders entered within 24 hours.^4^ Previous research has also shown that Black patients may have differing rates of DNAR order placement than White patients.^5^ It is unclear whether differences in DNAR order placement by race and ethnicity contribute to the survival gap after resuscitation from IHCA. The objective of this study was to understand whether American Indian or Alaskan Native, Black, or Hispanic patients have different rates of early DNAR orders compared with White patients, and to examine whether survival differences by race and ethnicity persist among patients with early entry of DNAR orders.

## Methods

This cohort study was declared not human participants research by the Yale University institutional review board and therefore was exempt from approval and informed consent. We followed the Strengthening the Reporting of Observational Studies in Epidemiology (STROBE) reporting guideline for cohort studies.

### Study Design and Setting

We used the American Heart Association’s Get With the Guidelines – Resuscitation (GWTG-R) dataset to conduct our study. The Get With The Guidelines programs are provided by the American Heart Association. The GWTG-R dataset includes prospectively collected IHCA data from more than 350 hospitals in the US in a quality improvement registry.^6^ Hospitals use the standardized Ustein definitions for variables for data entry.^7,8^ Hospitals participating in the registry submit clinical information regarding the medical history, hospital care, and outcomes of consecutive patients hospitalized for cardiac arrest using an online, interactive case report form and Patient Management Tool (IQVIA). Full details on the GWTG-R registry have been described in detail previously.^9,10,11^ We included patients with IHCA from 2018 to 2023.

### Study Population

We identified patients who were aged 18 years and older, experienced an index IHCA, and were successfully resuscitated while on an admitted unit. We excluded arrests that took place in the emergency department and other procedural units and patients who had a DNAR order placed before the cardiac arrest event based on time stamp of DNAR. We excluded hospitals with data for fewer than 10 patients per site, and we excluded patients with missing sex, event duration, or hospital characteristics.

### Study Variables

We included the following covariates in our adjusted models: year of event, age (18-49, 50-59, 60-69, 70-79, ≥80 years), race and ethnicity (categorized as American Indian or Alaskan Native, Asian, Native Hawaiian or Pacific Islander, Hispanic, non-Hispanic Black, non-Hispanic White, and unknown), Cerebral Performance Category (CPC) at admission^12^ (normal, moderate disability, severe disability, unconscious, or brain death), illness category (medical; cardiac and medical; noncardiac and surgical; cardiac and surgical; noncardiac, obstetric, trauma, or missing; or other), acute central nervous system nonstroke event, acute stroke, congestive heart failure diagnosed this admission, congestive heart failure diagnosed prior to admission, diabetes, hepatic insufficiency, hypotension or hypoperfusion, metastatic or hematologic malignant neoplasm, metabolic electrolyte abnormality, myocardial infarction prior admission, myocardial infarction this admission, pneumonia, renal insufficiency, respiratory insufficiency septicemia, any vasoactive agent in place at time of arrest, first pulseless rhythm (asystole, pulseless electrical activity, pulseless ventricular tachycardia, ventricular fibrillation, or unknown), ventricular fibrillation or pulseless ventricular tachycardia at any point during resuscitation, event witnessed, received chest compressions, duration of resuscitation prior to ROSC (0-5, 6-10, 11-20, or ≥21 minutes or missing), COVID-19 diagnosis (yes, prior to admission; yes, during this admission; no; or unknown), and time from ROSC to DNAR order, discharge, or death (hours). Hospital-level covariates included mean daily census of hospital (number of people) and region (Northeast, Midwest, South, West). All variables, including race and ethnicity, were abstracted at individual hospitals from electronic health record data.

### Missing Data

We anticipated missing CPC score data. We included patients missing CPC score as a separate category. We calculated Cardiac Arrest Survival Post-Resuscitation In-Hospital scores using established criteria that do not assign points for missing values. Missing binary variables (eg, renal insufficiency, mechanical ventilation) were coded using single imputation values of no. Missing multicategory variables (eg, arrest rhythm) were included as separate categories within covariates.

### Statistical Analysis

We used descriptive analyses to summarize frequencies and means for each covariate. We used generalized linear mixed-effects models with a logistic link function to answer study questions and adjusted for the covariates. Hospitals were included as random effects. The study outcome was not censored. In the first model, we evaluated the association of race and ethnicity with entry of early DNAR orders. We defined very early and early DNAR as within 12 or 72 hours after resuscitation, respectively. In the second model, we limited the dataset to include patients who had very early or early DNAR orders entered, and then evaluated the association of race and survival with hospital discharge. *P* values were 2-sided, and statistical significance was set at α = .05. All analyses were conducted in R software version 4.4.2 (R Project for Statistical Computing). Data were analyzed from September 26, 2024, through February 8, 2025.

## Results

From 2018 to 2023, we identified 93 843 patients (25 386 patients (27.1) aged 60-69 years; 56 533 [60.2%] male) who achieved ROSC after IHCA (Figure), including 2380 American Indian or Alaska Native patients (2.5%), 764 Asian patients (0.8%), 21 261 Black patients (22.7%), 6998 Hispanic patients (7.5%), 447 Native Hawaiian or Pacific Islander patients (0.5%), and 56 989 White patients (60.7%). Missing race and ethnicity data occurred in 5004 patients (5.3%). Descriptive data on the full study cohort by patient-reported race and ethnicity are in Table 1.

**Figure.**
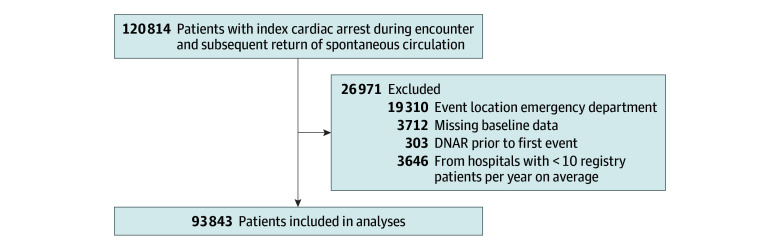
Study Cohort of Patients With In-Hospital Cardiac Arrest, 2018 to 2023 DNAR indicates do not attempt resuscitation.

**Table 1.** Demographics of a National Cohort of Patients With In-Hospital Cardiac Arrest, 2018 to 2023

Characteristic or category	Patients, No. (%)								*P* value
	Overall	American Indian or Alaska Native	Asian	Hispanic	Native Hawaiian or Pacific Islander	Non-Hispanic Black	Non-Hispanic White	Unknown	
Year of event									
2018	14 091 (15.0)	316 (13.3)	102 (13.4)	913 (13.0)	39 (8.7)	3186 (15.0)	8932 (15.7)	603 (12.1)	<.001
2019	15 400 (16.4)	342 (14.4)	109 (14.3)	1021 (14.6)	38 (8.5)	3462 (16.3)	9535 (16.7)	893 (17.8)	
2020	16 296 (17.4)	412 (17.3)	115 (15.1)	1335 (19.1)	78 (17.4)	3780 (17.8)	9507 (16.7)	1069 (21.4)	
2021	17 794 (19)	449 (18.9)	153 (20.0)	1419 (20.3)	97 (21.7)	4299 (20.2)	10 345 (18.2)	1032 (20.6)	
2022	16 022 (17.1)	451 (18.9)	151 (19.8)	1219 (17.4)	91 (20.4)	3499 (16.5)	9816 (17.2)	795 (15.9)	
2023	14 240 (15.2)	410 (17.2)	134 (17.5)	1091 (15.6)	104 (23.3)	3035 (14.3)	8854 (15.5)	612 (12.2)	
Age at event, y									
18-49	14 289 (15.2)	297 (12.5)	208 (27.2)	1682 (24.0)	101 (22.6)	4156 (19.5)	6866 (12.0)	979 (19.6)	<.001
50-59	15 115 (16.1)	299 (12.6)	164 (21.5)	1402 (20.0)	104 (23.3)	3878 (18.2)	8446 (14.8)	822 (16.4)	
60-69	25 386 (27.1)	559 (23.5)	214 (28.0)	1811 (25.9)	122 (27.3)	5972 (28.1)	15 344 (26.9)	1364 (27.3)	
70-79	24 460 (26.1)	718 (30.2)	137 (17.9)	1380 (19.7)	87 (19.5)	4723 (22.2)	16 247 (28.5)	1168 (23.3)	
≥80	14 593 (15.6)	507 (21.3)	41 (5.4)	723 (10.3)	33 (7.4)	2532 (11.9)	10 086 (17.7)	671 (13.4)	
Sex									
Male	56 533 (60.2)	1475 (62.0)	434 (56.8)	4379 (62.6)	264 (59.1)	11 598 (54.6)	35 200 (61.8)	3183 (63.6)	<.001
Female	37 310 (39.8)	905 (38.0)	330 (43.2)	2619 (37.4)	183 (40.9)	9663 (45.4)	21 789 (38.2)	1821 (36.4)	
Cerebral performance category at admission									
Normal (1)	53 151 (56.6)	1334 (56.1)	492 (64.4)	4048 (57.8)	275 (61.5)	11 086 (52.1)	33 191 (58.2)	2725 (54.5)	<.001
Moderate disability (2)	13 193 (14.1)	371 (15.6)	102 (13.4)	1068 (15.3)	58 (13)	3462 (16.3)	7594 (13.3)	538 (10.8)	
Severe disability (3)	8256 (8.8)	246 (10.3)	61 (8.0)	641 (9.2)	37 (8.3)	2417 (11.4)	4453 (7.8)	401 (8.0)	
Unconscious (4)	5355 (5.7)	141 (5.9)	47 (6.2)	394 (5.6)	30 (6.7)	1345 (6.3)	2949 (5.2)	449 (9.0)	
Brain death	82 (0.1)	4 (0.2)	1 (0.1)	4 (0.1)	1 (0.2)	26 (0.1)	38 (0.1)	8 (0.2)	
Missing	13 806 (14.7)	284 (11.9)	61 (8.0)	843 (12.0)	46 (10.3)	2925 (13.8)	8764 (15.4)	883 (17.6)	
Illness category									
Medical-cardiac	31 130 (33.2)	814 (34.2)	228 (29.8)	2256 (32.2)	194 (43.4)	6411 (30.2)	19 463 (34.2)	1764 (35.3)	<.001
Medical-noncardiac	44 475 (47.4)	1121 (47.1)	416 (54.5)	3629 (51.9)	191 (42.7)	11 642 (54.8)	25 309 (44.4)	2167 (43.3)	
Surgical-cardiac	6259 (6.7)	165 (6.9)	30 (3.9)	352 (5.0)	27 (6)	854 (4)	4459 (7.8)	372 (7.4)	
Surgical-noncardiac	9060 (9.7)	231 (9.7)	67 (8.8)	563 (8.0)	24 (5.4)	1733 (8.2)	5997 (10.5)	445 (8.9)	
Obstetric	159 (0.2)	11 (0.5)	3 (0.4)	29 (0.4)	1 (0.2)	48 (0.2)	59 (0.1)	8 (0.2)	
Trauma		34 (1.4)	17 (2.2)	161 (2.3)	8 (1.8)	541 (2.5)	1597 (2.8)	231 (4.6)	
Other or missing		4 (0.2)	3 (0.4)	8 (0.1)	2 (0.4)	32 (0.2)	105 (0.2)	17 (0.3)	
Acute central nervous system nonstroke event	12 627 (13.5)	306 (12.9)	125 (16.4)	842 (12.0)	51 (11.4)	3192 (15.0)	7489 (13.1)	622 (12.4)	<.001
Acute stroke	4252 (4.5)	128 (5.4)	30 (3.9)	302 (4.3)	23 (5.1)	1218 (5.7)	2314 (4.1)	237 (4.7)	<.001
CHF diagnosed this admission		328 (13.8)	116 (15.2)	963 (13.8)	78 (17.4)	3024 (14.2)	8216 (14.4)	613 (12.3)	<.001
CHF diagnosed prior admission	24 377 (26.0)	545 (22.9)	193 (25.3)	1663 (23.8)	155 (34.7)	6306 (29.7)	14 520 (25.5)	995 (19.9)	<.001
Diabetes	36 706 (39.1)	1110 (46.6)	345 (45.2)	3547 (50.7)	250 (55.9)	9330 (43.9)	20 457 (35.9)	1667 (33.3)	<.001
Hepatic insufficiency	10 108 (10.8)	300 (12.6)	129 (16.9)	1155 (16.5)	57 (12.8)	2351 (11.1)	5538 (9.7)	578 (11.6)	<.001
Hypotension/hypoperfusion	28 990 (30.9)	788 (33.1)	248 (32.5)	2301 (32.9)	162 (36.2)	6929 (32.6)	17 066 (29.9)	1496 (29.9)	<.001
Metastatic or hematologic malignant neoplasm	10 388 (11.1)	283 (11.9)	57 (7.5)	581 (8.3)	33 (7.4)	2602 (12.2)	6342 (11.1)	490 (9.8)	<.001
Metabolic electrolyte abnormality	27 408 (29.2)	824 (34.6)	239 (31.3)	2398 (34.3)	184 (41.2)	6852 (32.2)	15 503 (27.2)	1408 (28.1)	<.001
MI prior admission	13 630 (14.5)	289 (12.1)	116 (15.2)	776 (11.1)	63 (14.1)	2562 (12.1)	9235 (16.2)	589 (11.8)	<.001
M this admission	13 175 (14.0)	383 (16.1)	110 (14.4)	813 (11.6)	94 (21)	2078 (9.8)	8987 (15.8)	710 (14.2)	<.001
Pneumonia	18 794 (20.0)	495 (20.8)	195 (25.5)	1691 (24.2)	100 (22.4)	4662 (21.9)	10 753 (18.9)	898 (17.9)	<.001
Renal insufficiency	36 841 (39.3)	1048 (44.0)	328 (42.9)	3335 (47.7)	263 (58.8)	10 924 (51.4)	19 166 (33.6)	1777 (35.5)	<.001
Respiratory insufficiency	48 066 (51.2)	1206 (50.7)	359 (47.0)	3778 (54.0)	226 (50.6)	11 672 (54.9)	28 297 (49.7)	2528 (50.5)	<.001
Septicemia	1403 (1.5)	36 (1.5)	12 (1.6)	112 (1.6)	2 (0.4)	405 (1.9)	777 (1.4)	59 (1.2)	<.001
Any vasoactive agent in place	28 410 (30.3)	800 (33.6)	260 (34.0)	2359 (33.7)	161 (36)	6391 (30.1)	16 654 (29.2)	1785 (35.7)	<.001
Induced hypothermia after arrest									
Yes	8059 (8.6)	238 (10.0)	132 (17.3)	643 (9.2)	67 (15.0)	1839 (8.6)	4684 (8.2)	456 (9.1)	<.001
No, not documented, or missing	85 784 (91.4)	2142 (90.0)	632 (82.7)	6355 (90.8)	380 (85.0)	19 422 (91.4)	52 305 (91.8)	4548 (90.9)	
First pulseless rhythm									
Asystole	19 355 (20.6)	553 (23.2)	131 (17.1)	1446 (20.7)	78 (17.4)	4355 (20.5)	11 795 (20.7)	997 (19.9)	<.001
PEA	50 868 (54.2)	1308 (55.0)	416 (54.5)	4031 (57.6)	234 (52.3)	12 751 (60.0)	29 426 (51.6)	2702 (54.0)	
pVT	7873 (8.4)	175 (7.4)	82 (10.7)	448 (6.4)	38 (8.5)	1194 (5.6)	5484 (9.6)	452 (9.0)	
VF	7726 (8.2)	157 (6.6)	55 (7.2)	462 (6.6)	35 (7.8)	1205 (5.7)	5393 (9.5)	419 (8.4)	
Unknown, not documented, or missing	8021 (8.5)	187 (7.9)	80 (10.5)	611 (8.7)	62 (13.9)	1756 (8.3)	4891 (8.6)	434 (8.7)	
VF or pVT at any point	27 179 (29.0)	653 (27.4)	232 (30.4)	1770 (25.3)	132 (29.5)	5174 (24.3)	17 703 (31.1)	1515 (30.3)	<.001
Event witnessed	83 435 (88.9)	2166 (91.0)	692 (90.6)	6240 (89.2)	411 (91.9)	18 918 (89.0)	50 469 (88.6)	4539 (90.7)	<.001
Received chest compressions	92 921 (99.0)	2362 (99.2)	757 (99.1)	6948 (99.3)	446 (99.8)	21 159 (99.5)	56 292 (98.8)	4957 (99.1)	<.001
Assisted or mechanical ventilation	60 (0.1)	5 (0.2)	0	5 (0.1)	0	33 (0.2)	14 (<0.1)	3 (0.1)	<.001
State category									
Midwest	19 603 (20.9)	271 (11.4)	116 (15.2)	506 (7.2)	25 (5.6)	3830 (18.0)	14 010 (24.6)	845 (16.9)	<.001
Northeast	15 061 (16.0)	433 (18.2)	52 (6.8)	1582 (22.6)	6 (1.3)	2960 (13.9)	8994 (15.8)	1034 (20.7)	
South	42 995 (45.8)	532 (22.4)	276 (36.1)	1884 (26.9)	44 (9.8)	13 268 (62.4)	25 694 (45.1)	1297 (25.9)	
West	16 184 (17.2)	1144 (48.1)	320 (41.9)	3026 (43.2)	372 (83.2)	1203 (5.7)	8291 (14.5)	1828 (36.5)	
Event duration, min									
0-5	33 807 (36.0)	842 (35.4)	262 (34.3)	2496 (35.7)	143 (32)	7160 (33.7)	21 159 (37.1)	1745 (34.9)	<.001
6-10	21 917 (23.4)	554 (23.3)	175 (22.9)	1709 (24.4)	132 (29.5)	5187 (24.4)	13 041 (22.9)	1119 (22.4)	
11-20	18 529 (19.7)	482 (20.3)	178 (23.3)	1397 (20.0)	91 (20.4)	4349 (20.5)	11 055 (19.4)	977 (19.5)	
≥21	16 113 (17.2)	447 (18.8)	137 (17.9)	1183 (16.9)	77 (17.2)	3823 (18.0)	9473 (16.6)	973 (19.4)	
Missing	3477 (3.7)	55 (2.3)	12 (1.6)	213 (3.0)	4 (0.9)	742 (3.5)	2261 (4.0)	190 (3.8)	
Daily inpatient census of hospital, mean (SD), No.	452.74 (0.96)	428.28 (273.73)	397.44 (250.06)	430.81 (255.92)	334.26 (202.64)	496.51 (312.08)	441.71 (292.01)	453.79 (264.88)	<.001
COVID-19 diagnosis									
Yes, prior to admission	3997 (4.3)	98 (4.1)	47 (6.2)	507 (7.2)	27 (6.0)	952 (4.5)	2114 (3.7)	252 (5)	<.001
Yes, during hospitalization	3858 (4.1)	102 (4.3)	21 (2.7)	563 (8.0)	13 (2.9)	1095 (5.2)	1846 (3.2)	218 (4.4)	
No	39 799 (42.4)	966 (40.6)	363 (47.5)	2826 (40.4)	157 (35.1)	8792 (41.4)	24 737 (43.4)	1958 (39.1)	
Unknown	2738 (2.9)	54 (2.3)	24 (3.1)	172 (2.5)	14 (3.1)	680 (3.2)	1585 (2.8)	209 (4.2)	
Missing	43 451 (46.3)	1160 (48.7)	309 (40.4)	2930 (41.9)	236 (52.8)	9742 (45.8)	26 707 (46.9)	2367 (47.3)	
Survival to hospital discharge	34 768 (37.0)	7168 (33.7)	813 (34.2)	22 226 (39.0)	273 (35.7)	2417 (34.5)	23 897 (41.9)	178 (39.8)	<.001
DNAR established, h									
<12	22 370 (23.8)	508 (21.3)	178 (23.3)	1554 (22.2)	113 (25.3)	4560 (21.4)	14 417 (25.3)	1040 (20.8)	<.001
12-72	11 047 (11.8)	288 (12.1)	106 (13.9)	772 (11.0)	48 (10.7)	2388 (11.2)	6907 (12.1)	538 (10.8)	
>72	13 267 (14.1)	382 (16.1)	97 (12.7)	1042 (14.9)	62 (13.9)	3249 (15.3)	7751 (13.6)	684 (13.7)	
Not established	47 159 (50.3)	1202 (50.5)	383 (50.1)	3630 (51.9)	224 (50.1)	11 064 (52)	27 914 (49.0)	2742 (54.8)	
CASPRI score									
0-9	16 840 (17.9)	31.00 (13)	154 (20.2)	1099 (15.7)	84 (18.8)	2814 (13.2)	11 446 (20.1)	933 (18.6)	<.001
>9	77 003 (82.1)	2070 (87.0)	610 (79.8)	5899 (84.3)	363 (81.2)	18 447 (86.8)	45 543 (79.9)	4071 (81.4)	
Time from ROSC to DNAR order, discharge, or death, mean (SD), h	75.98 (72.9)	79.04 (73.65)	77.07 (72.18)	78.98 (74.02)	80.65 (74.12)	77.79 (73.63)	74.76 (72.41)	76.04 (73.16)	<.001

Abbreviations: CASPRI, Cardiac Arrest Survival Post-Resuscitation In-Hospital; CHF, congestive heart failure; DNAR, do not attempt resuscitation; MI, myocardial infarction; PEA, pulseless electrical activity; pVT, pulseless ventricular tachycardia; ROSC, return of spontaneous circulation; VF, ventricular fibrillation.

After resuscitation from cardiac arrest, 25.3% and 37.4% of White patients had DNAR orders entered at 12 hours and 72 hours, respectively, compared with 21.3% and 33.4% of American Indian or Alaska Native patients, 21.4% and 32.7% of Black patients, and 22.2% and 33.2% of Hispanic patients (Table 2). In our adjusted models, American Indian or Alaska Native, Black, and Hispanic patients were less likely to have DNAR orders entered within 12 hours (American Indian or Alaska Native: OR, 0.78 [95% CI, 0.67-0.91]; Black: OR, 0.74 [95% CI, 0.69-0.79]; Hispanic: OR, 0.90 [95% CI, 0.82-0.99]) or within 72 hours (American Indian or Alaska Native: OR, 0.86 [95% CI, 0.76-0.98]; Black: OR, 0.73 [95% CI, 0.69-0.77]; Hispanic: OR, 0.89 [95% CI, 0.83- 0.97]) than White patients.

**Table 2.** Race/Ethnicity and Early DNAR Orders Among a National Cohort of Patients With In-Hospital Cardiac Arrest, 2018 to 2023

Race and ethnicity	DNAR order timing						
	Patients, No. (%)			OR (95% CI)			
				<12 h		<72 h	
	<12 h	<72 h	None	Unadjusted	Adjusted	Unadjusted	Adjusted
American Indian or Alaska Native^a^	508 (2.27)	796 (2.38)	1584 (2.62)	0.83 (0.74-0.92)	0.78 (0.67-0.91)	0.88 (0.80-0.97)	0.86 (0.76-0.98)
Asian^b^	178 (0.80)	284 (0.85)	480 (0.79)	0.88 (0.73-1.05)	1.03 (0.79-1.35)	1.00 (0.85-1.17)	1.14 (0.91-1.43)
Hispanic^a^	1554 (6.95)	2326 (6.96)	4672 (7.73)	0.87 (0.82-0.93)	0.90 (0.82-0.99)	0.86 (0.81-0.91)	0.89 (0.83-0.97)
Native Hawaiian or Pacific Islander^b^	113 (0.51)	161 (0.48)	286 (0.47)	0.91 (0.72-1.15)	1.15 (0.80-1.65)	0.89 (0.72-1.11)	1.13 (0.83-1.54)
Non-Hispanic-Black^a^	4560 (20.38)	6948 (20.79)	14 313 (23.69)	0.80 (0.77-0.83)	0.74 (0.69-0.79)	0.80 (0.77-0.83)	0.73 (0.69-0.77)
Non-Hispanic-White	14 417 (64.45)	21 324 (63.81)	35 665 (59.02)	1 [Reference]	1 [Reference]	1 [Reference]	1 [Reference]
Unknown^a^	1040 (4.65)	1578 (4.72)	3426 (5.67)	0.85 (0.79-0.92)	0.79 (0.71-0.87)	0.87 (0.81-0.93)	0.81 (0.74-0.88)

Abbreviations: DNAR, do not attempt resuscitation; OR, odds ratio.

^a^ Analyses had consistent significance across all 4 analyses.

^b^ Analyses were consistently not statistically significant across all 4 analyses.

Over the course of the study, 22 226 White patients (39.0%) survived to hospital discharge, compared with 813 American Indian or Alaska Native patients(34.2%), 7168 Black patients (33.7%), and 2417 Hispanic patients (34.5%). In adjusted analyses, among patients with an early DNAR order entered before 72 hours, there was no significant difference in survival to hospital discharge compared with White patients (Table 3).

**Table 3.** Survival to Hospital Discharge Among Patients With Early Do Not Attempt Resuscitation Orders Within a National Cohort of Patients With In-Hospital Cardiac Arrest, 2018-2023

Race and ethnicity	Patients, No. (%)		Survival to hospital discharge, OR (95% CI)	
	Survived	Died	Unadjusted	Adjusted
American Indian or Alaska Native	813 (2.34)	1565 (2.65)	0.74 (0.54-1.01)	0.77 (0.56-1.06)
Asian	273 (0.79)	489 (0.83)	0.78 (0.47-1.30)	0.91 (0.54-1.54)
Hispanic	2417 (6.95)	4581 (7.76)	0.72 (0.60-0.88)	0.82 (0.67-1.00)
Native Hawaiian or Pacific Islander	178 (0.51)	269 (0.46)	0.57 (0.27-1.21)	0.75 (0.34-1.65)
Non-Hispanic Black	7168 (20.62)	14 089 (23.86)	0.87 (0.78-0.98)	0.98 (0.86-1.10)
Non-Hispanic White	22 226 (63.93)	34 751 (58.85)	1 [Reference]	1 [Reference]
Unknown	1693 (4.87)	3311 (5.61)	0.72 (0.57-0.90)	0.83 (0.66-1.05)

Abbreviations: NA, not applicable; OR, odds ratio.

## Discussion

In this large national cohort study of patients with IHCA, early DNAR rates were high: 1 in 5 patients had a DNAR order placed in the 12 hours after ROSC, and 1 in 3 had a DNAR order placed in the 72 hours after ROSC. The high rates of DNAR order placement in our study are similar to what has previously been reported. GWTG data from 2006 to 2012 found that 22.5% of patients had a DNAR order entered in the first 12 hours after cardiac arrest.^13^ In this analysis of data from 2018 to 2023, the rate of early DNAR orders within 12 hours of cardiac arrest was similar to 2006 to 2012 data, despite guidelines in the interim that endorse waiting at least 72 hours from ROSC to perform neuroprognostication.^14,15^ Outside of GWTG-R, little data have explored early DNAR rates after cardiac arrest. After out-of-hospital cardiac arrest in Michigan, 14.5% of patients had a DNAR order placed within 72 hours of hospital admission across 38 hospitals.^16^

American Indian or Alaska Native, Black, and Hispanic patients were less likely to have early DNAR orders placed than White patients. Our results map onto earlier research that Black patients are less likely to have advanced directives prior to hospitalization or to choose DNAR orders once hospitalized compared with White patients^17,18,19,20^; the reasons for this are complex but likely include cultural, social, and religious factors, as well as mistrust of health care systems.^21,22,23^ Evidence for Hispanic patients tends to conflict: while some studies find lower rates of DNAR among Hispanic patients,^17^ others found rates higher than those of White patients.^20^ Previous research among American Indian or Alaska Native patients reported that some tribes may be less willing to discuss death for fear that discussing death will bring it on^24,25^; however, American Indian or Alaska Native communities are not homogenous in the US and have specific cultural, family, and interpersonal values that intersect with a unique health care system, the Indian Health Service.^26,27,28^ Culturally specific research could further explore how decisions about DNAR orders are made following cardiac arrest for American Indian or Alaska Native, Black, and Hispanic patients.

Among patients with early DNAR orders placed, there was no significant survival difference by race or ethnicity. Therefore, it is unlikely that the placement of early DNAR orders in isolation drives a disparity in survival to hospital discharge among variable racial and ethnic groups. One might surmise that if White patients have a higher incidence of DNAR, they should equally experience less survival, yet no survival difference was measured. One study described a recent improvement in survival of Black patients to hospital discharge after IHCA, specifically outlining a greater measurement of survival at hospitals with a higher proportion of Black patients.^29^ Previous research has also found that resolution of disparities in nursing staffing and hospital resources improved survival after IHCA for Black patients.^2,30^ Differences in survival to hospital discharge in Black patients compared with White patients are likely multifactorial, but our analysis found that there were no differences by race and ethnicity among patients with early DNAR orders placed.

### Limitations

This study has some limitations. Our research is limited by the use of a quality assurance dataset that does not provide insight into how decisions are made about early DNAR orders, which limits our understanding of why early DNAR orders are deployed. Additional qualitative research is needed to understand decision-making among families and clinicians. Race and ethnicity were missing in a proportion of patients; therefore, they were excluded from this analysis. Exploring ways to improve capture of important demographic data is necessary to fully understand complex scenarios in observational data. While GWTG-R is a comprehensive collection of data from more than 350 US hospitals, it is a sample of patients who experience IHCA and may not be reflective of the population as a whole.

## Conclusions

In this cohort study, American Indian or Alaska Native, Black, and Hispanic patients were less likely to have early DNAR orders entered than White patients, but there were no differences in survival by race among patients with early DNAR orders placed. More research is needed to understand how families and clinicians make decisions about DNAR orders after resuscitation from in-hospital cardiac arrest.

## Notes

Supplement.
Data Sharing Statement
