# Supplementary material for: Race and Ethnicity and Early Do Not Attempt Resuscitation Orders After In-Hospital Cardiac Arrest
Source: JAMA Netw Open. 2026 Jan 13;9(1):e2553504. doi: 10.1001/jamanetworkopen.2025.53504 (PMC12801091; doi:10.1001/jamanetworkopen.2025.53504)
Supplement: Supplement. — Data Sharing Statement [file jamanetwopen-e2553504-s001.pdf]

Data Sharing Statement

Raymond-King. Race and Ethnicity and Early Do Not Attempt Resuscitation Orders After In-Hospital Cardiac Arrest. *JAMA Netw Open*. Published January 13, 2026.
doi:10.1001/jamanetworkopen.2025.53504

Data
Data available: No

Additional Information
Explanation for why data not available: Data are available through the Get With the Guidelines-Resuscitation committee.
